# Supplementary material for: PhCHS5 and PhF3′5′H Genes Over-Expression in Petunia (Petunia hybrida) and Phalaenopsis (Phalaenopsis aphrodite) Regulate Flower Color and Branch Number
Source: Plants (Basel). 2023 Jun 2;12(11):2204. doi: 10.3390/plants12112204 (PMC10255828; doi:10.3390/plants12112204)
Supplement: Supplementary file 1 [file plants-12-02204-s001.zip › plants-2304838-supplementary.pdf]

Supplementary File

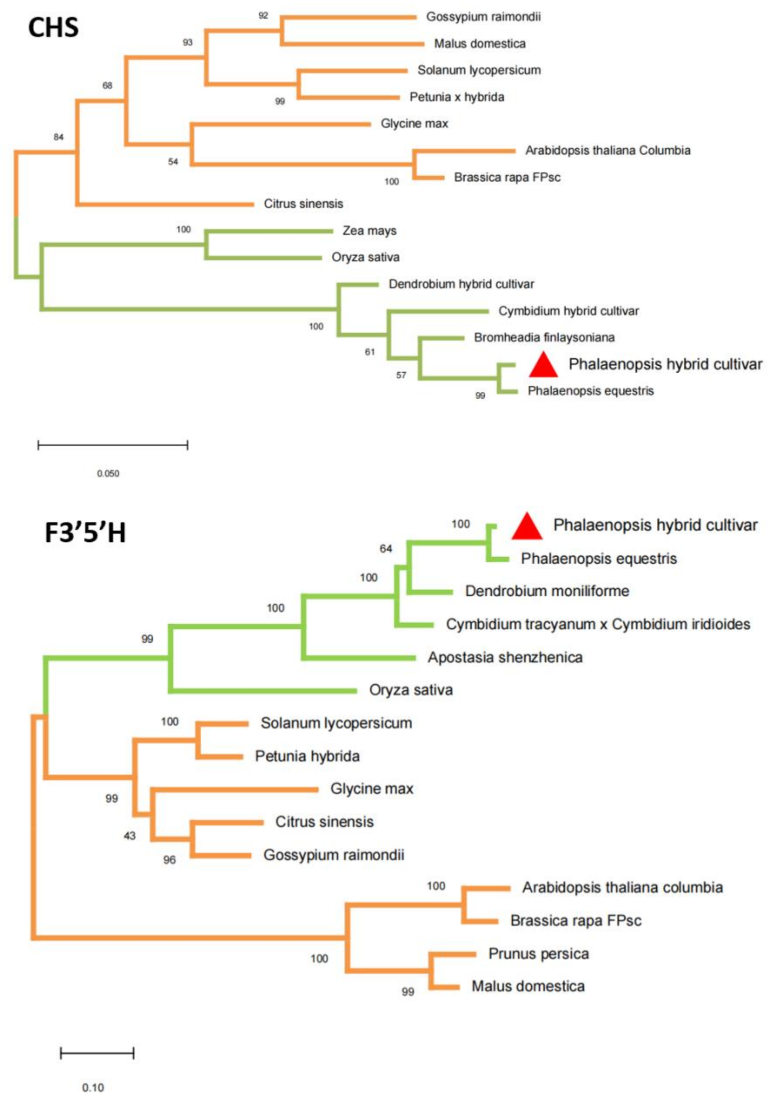

Fig. S1 Phylogenetic analysis of CHS5 and F3'5'H in different plant species. (a) CHS5; (b) F3'5'H.

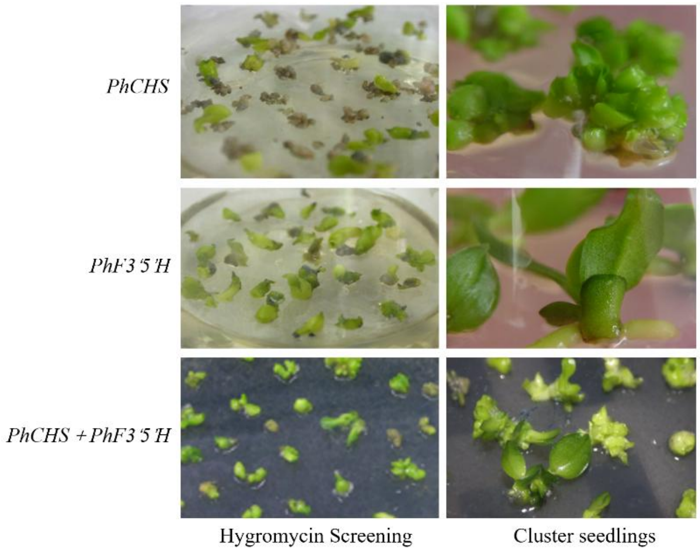

Fig. S2 Hygromycin screening and cluster seedlings of the transgenic *Phalaenopsis* samples.

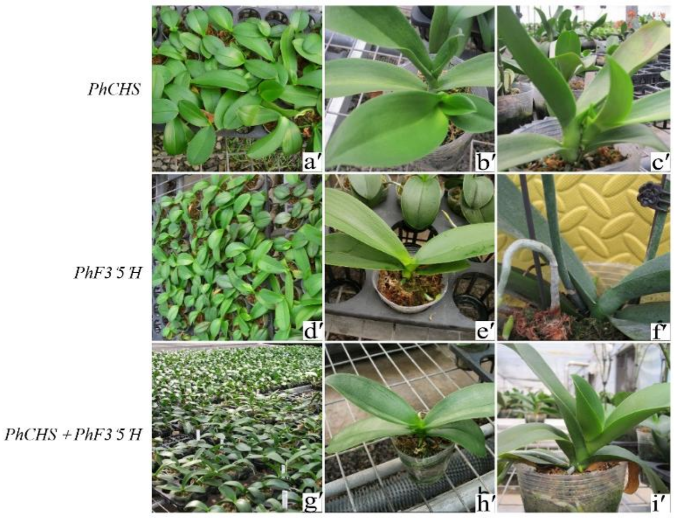

Fig. S3 Leaf phenotypes of the transgenic *Phalaenopsis* samples. (a′–c′) *PhCHS5*-transformed plants. (d′–f′) *PhF3'5'H*-transformed plants. (g′–i′) *PhCHS5* + *PhF3'5'H*-transformed plants.

Table S1 Medium information for protocorm-likebodies（PLB）induction and differentiation and Agrobacterium infection

Name	function	medium gradients	notes
YD1	PLBs induction	MS 4.4g/L+ BA(ppm)/NAA(ppm) 2.0/0.2+ 20g/L sucrose+CH 500mg/L +AC 50mg/ml	active carbon（AC）
YD 2	PLBs induction	MS 4.4g/L+ BA(ppm)/NAA(ppm) 2.0/2.0+ 20g/L sucrose+CH 500mg/L +AC 50mg/ml	casein hydrolysate(CH)
YD 3	PLBs induction	MS 4.4g/L+ BA(ppm)/NAA(ppm) 3.0/0.1+ 20g/L sucrose+CH 500mg/L +AC 50mg/ml	solid medium with

YD4	PLBs induction	MS 4.4g/L+ BA(ppm)/NAA(ppm) 3.0/2.0+ 20g/L sucrose+CH 500mg/L +AC 50mg/ml	phytagel 2.7g/L pH=6.2
YDR	PLBs Rooting	MS 2.2g/L +IBA(0.1mg/ml)+sucrose 30g/L+CH 500mg/L + AC（50mg/ml）	
YD3T	transformation medium solution for *Agrobacterium* infection	MS 4.4g/L+ BA(ppm)/NAA(ppm) 3.0/0.1+ 20g/L sucrose+CH 500mg/L +AC 50mg/ml	Without phytagel and AC , with As 100mg/l
